# Characterization of the Earwig, *Doru lineare*, as a Predator of Larvae of the Fall Armyworm, *Spodoptera frugiperda*: A Functional Response Study

**DOI:** 10.1673/031.010.3801

**Published:** 2010-04-27

**Authors:** Mabel Romero Sueldo, Octavio A. Bruzzone, Eduardo G. Virla

**Affiliations:** ^1^ Sección Entomología, Fundación Miguel Lillo. M. Lillo 250 3er piso (CP 4000), S.M. de Tucumán, Tucumán, Argentina; ^2^ Laboratorio de Ecología de lnsectos, INTA, EEA Bariloche. (8400) Bariloche, Rio Negro, Argentina; ^3^ PROIMI-Biotecnología, Div. Control Biológico, Av. Belgrano y Pje. Caseros (T4001 MVB), San Miguel de Tucumán, Tucumán, Argentina

**Keywords:** behavior, biological control, handling time, predation efficiency

## Abstract

*Spodoptera frugiperda* Smith (Lepidoptera: Noctuidae) is considered as the most important pest of maize in almost all tropical America. In Argentina, the earwig *Doru lineare* Eschscholtz (Dermaptera: Forficulidae) has been observed preying on *S. frugiperda* egg masses in corn crops, but no data about its potential role as a biocontrol agent of this pest have been provided. The predation efficiency of *D. lineare* on newly emerged *S. frugiperda* larva was evaluated through a laboratory functional response study. *D. lineare* showed type II functional response to *S. frugiperda* larval density, and disc equation estimations of searching efficiency and handling time were (a) = 0.374 and (t) = 182.9 s, respectively. Earwig satiation occurred at 39.4 *S. frugiperda* larvae.

## Introduction

The fall armyworm, *Spodoptera frugiperda* Smith (Lepidoptera: Noctuidae), is an important pest of many crops, causing important yield losses in different regions of the Americas (Sparks 1979). In northern Argentina, *S. frugiperda* infestations may result in maize yield losses between 17% and 72% (Perdiguero et al. 1967). To date, control of this pest relied mainly on the use of synthetic pesticides (Hruska and Gould 1997), and as a result, individuals resistant to insecticides have been selected (Yu et al. 2003). Resistance of *S. frugiperda* to various carbamate, organophophorus, and pyrethroid insecticides has also been observed in field strains collected from corn in north, central and south Florida (Yu 1991, 1992).

*S. frugiperda* is a native pest in Argentina with a diverse complex of natural enemies. The impact of parasitoids on these populations has been well studied (Virla et al. 1999; Berta et al. 2000; Murua et al. 2003). In turn, some work has focused on entomopathogenic organisms such as nuclear polyhedrosis virus, *Nomuraea rileyi*, *Metahrizium anisopliae* and *Beauveria bassiana* (see references in Vera et al. 1995; Berreta et al. 1998; Lecuona and Diaz 2001). However, there is a remarkable lack of information on the role of natural predators. A few exceptions are technical reports on the action against this pest by some carabid beetles and other Coleoptera, lacewings (Neuroptera), and some bugs (Heteroptera) (Saini 2005).

Dermaptera are omnivorous insects that may be considered either damaging or helpful organisms within agroecosystems (Van Huis 1981; Jones et al. 1988; Gravena and Da Cunha 1991; Mariani et al. 1996). The beneficial actions of Dermaptera in many crops of economical relevance have been described previously by Buxton (1974) and Cañellas et al. (2005). Most adult earwigs hide during the day in dark, but some species, like *Chelisoches morio*, are diurnal and extremely active, running over the leaves during the hottest part of the day in search of food (Zimmerman 1948, as cited by Hudson 1974).

*Doru taeniatum* has been reported as an effective predator of *S. frugiperda* in Central America (Jones et al. 1988; Lastres 1990). In Brazil, the importance of two species of Dermaptera in annual crops is well known: *Doru luteipes* seems to be the most abundant earwig and has been mentioned as an efficient predator of *S. frugiperda* and *Helicoverpa zea* in soybean (Lanza Reis et al. 1988; Cruz 1992, 1995); *Doru lineare* (Dermaptera: Forficulidae) also has been mentioned as a beneficial agent in soybean (Otero and Belarmino 1993, Belarmino and Gati 1993) and for controlling *Alabama argillacea* in cotton (Gravena and Da Cuhna 1991), *Diatraea saccharalis* in sugar cane (Soussa-Silva et al. 1992), and *Sitotroga cerealella* in stored grains.

Both *D. luteipes* and *D. lineare* species have been found in maize crops in northern Argentina, although their role in the agroecosystem is still unknown. In Tucumán province, during summertime, *D. lineare* show evidence of foraging activities through the day. This earwig previously was reported preying on *S. frugiperda* egg masses in corn crops (Mariani et al. 1996). Although some bionomic studies on *D. lineare* have been conducted under laboratory conditions (Romero Sueldo and Dode 2001), there is a lack of information about its predation-capability on *S. frugiperda* eggs and newly emerged larvae.

Recently, functional response of parasitoids and predators in relation to prey density has received increasing attention in the entomological literature (Houck and Strauss 1985; Mohaghegh et al. 2001; Fernández-Arhex and Corley 2003, 2004). However, there are no reports on the functional response of any Dermaptera species.

Because *S. frugiperda* is considered a key pest of corn in northwestern Argentina and because *D. lineare* populations frequently occur in the field, it needs to be determined if this species acts as a predator and should be considered a potential biological control agent. The aim of this study was to investigate the predation efficiency of this earwig through its functional response to *S. frugiperda* newly emerged larvae in the laboratory.

## Materials and Methods

### Origin and maintenance of insect colonies

Both *S. frugiperda* and *D. lineare* colonies were established with specimens collected during December 2004 from a subsistence cornfield located near El Manantial (Dpto. Lules, Tucuman province, Argentina) (26°49′50.2″S, 65°16′59.4″W, elevation: 495 m).

*S. frugiperda* larvae were placed individually in glass tubes (12 cm high × 1.5 cm diameter) with host leaves and carried to the laboratory. Adults of *S. frugiperda* were maintained in polyethylene-terephthalate cylindrical cages (30 cm high × 10 cm diameter). For aeration, the top was covered with a nylon mesh cloth. These cages contained pieces of paper that allowed females to rest and to lay eggs. Food was provided via a cotton wick saturated with a honey and water solution (1:1 vol/vol). Cages were checked daily for egg masses, and these were collected and deposited in glass tubes as above. Upon eclosion the neonate larvae were placed in 250 cc plastic pots containing artificial diet (Ozores et al. 1982). Pots were covered with a nylon mesh cloth until the larva reached the 3^rd^ instar, at which time they were isolated in glass tubes to prevent cannibalism.

Earwig colonies were maintained in plastic cages (30 × 25 × 8cm) containing pieces of corrugated cardboard as refuge. Commercial cat food and a cotton wick saturated with a honey + water solution (1:1 vol/vol) were provided as food. In each cage, a maximum of 20 couples were maintained together to prevent cannibalism. Cages were examined daily, and eggs were transferred carefully with the female to a 250 cc plastic pot and provided with a plastic soda cup filled with wet cotton (1.5 cm high - 3.0 cm diameter). Normally, females transported their offspring into the soda cups. Ten days after nymphal eclosion, they were transferred to larger plastic cages (as described above) until they completed development.

Insect cultures were conducted in the laboratory at 26 ± 2°C, 14:10 (L:D) photoperiod, and 70 ± 10% RH. All predatory individuals used in the experiments were reproductively active females of *D. lineare* that were two to three weeks old. Females were starved for 48 h before trials and were randomly collected from the breeding cages.

### Handling time trials

Handling time of newly emerged *S. frugiperda* larvae is defined in this study as the time interval starting with the piercing of larval tegument until the complete consumption of the prey item, excluding the cephalic capsule. Preliminary observations showed that sometimes earwigs do not consume the cephalic capsule.

Handling time was assessed by direct observation under stereoscopic microscope and measured with chronometer. Each female of *D. lineare* (*n* = 100) was placed with 5 to 10 larvae in a 6 cm (diameter) by 0.5 cm (depth) Petri dish. The trials were run at 26 ± 2°C.

Potted corn plants (2^nd^ vegetative stage) covered with a polyethylene-terephthalate cylindrical cages (35 cm long × 18 cm diameter) were used as the experimental arenas. Each cage was covered with a fine nylon mesh allowing air exchange.

### Functional response trials

Predation rate was determined by releasing a single female earwig on potted corn plants that contained newly emerged *S. frugiperda* larvae at different densities: 1, 7, 10, 20, 40, 70, 100, 115, 130, 160, 190, 250, 360, 420 and 500 larvae. Larvae were placed using a paintbrush in the whorl region, and usually most of them spread over the entire plant. So, at the release time, the prey were randomly distributed on the corn plant. After two hours, the predators were removed and the number of remaining intact larvae alive was recorded. Six replicates were done for each prey density, and consumed prey were not replaced.

Predator searching efficiency was obtained from the quantity of dead and available prey using the formula:

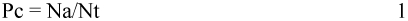

where, Pc = searching efficiency, Na = number of consumed prey, and Nt = number of offered prey.

### Data analysis

Following Trexler et al. (1988) and Fernández-Arhex and Corley (2004), a stepwise logistic regression (Legendre and Legendre 1998) was used to fit the best curve, where the functional responses of type II and III are differentiated by the presence of a different number of significant components in the *z* term of the equation:

Where *P* is the proportion of prey killed, *z* is the function of the prey abundance *x*, and *b_0–2_* are the parameters of function *z*.

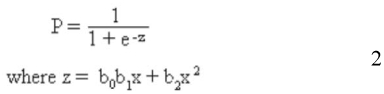

By this method, three different results are expected: (1) if none of the parameters, *b_0_*, *b_1_*, *b_2_* or only *b_0_* is significant, there is functional response type I in which the asymptote is not reached in the densities used in the experiment; (2) if b*_1_* is significantly negative, a type II functional response exists; and (3) if *b_1_* and *b_2_* are significantly positive and negative respectively, a type III functional response exists. Functional response type 1 was not considered since it does not make biological sense for insect predators.

The logistic model was fit to the data by stepwise nonlinear regression, using the maximum likelihood criterion and the Wald statistic (Legendre and Legendre 1998). The significance was calculated by comparing the Wald statistic with a *X^2^* distribution. The parameters were fitted iteratively using a program written in the Python programming language version 2.3.5 (Python Software Foundation 2005, www.python.org/psf/), with the numarray module for statistical analysis (Greenfield et al. 2003).

After selecting the type of functional response, the data were fit by nonlinear regression to one of the following equations: Type II “Disc Equation” or Type III (Hassell 2000). The regression was performed with the GNUPLOT version 4.0.0 program (Williams et al. 2004), using the weighted least squares method and statistical significance estimated by a *t*-test.

### Voucher specimens

Insect voucher specimens were deposited in the Instituto Fundación “Miguel Lillo” collection, San Miguel de Tucumán, Argentina.

## Results and Discussion

### Handling time trials

The observed time required for an earwig to consume a single, newly emerged *S. frugiperda* larvae ranged from 10.1 to 55.2 seconds (x = 24.3; SE = 8.8). Based on this mean handling time alone, maximum consumption was predicted to not exceed 296 *S. frugiperda* larvae in the 2 h functional response experiments.

### Functional response trials

**Figure 1.**
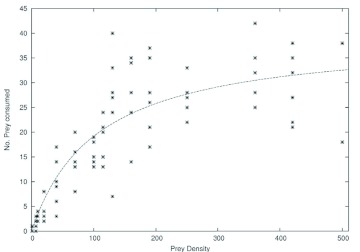
Functional response of *Doru lineare* females to different densities of first instar *Spodoptera frugiperda* larvae provided on corn plants in the laboratory. High quality figures are available online.

Logistic regression analyses revealed a type II functional response by *D. lineare* to *S. frugiperda* larval density (Figure 1). The Type II: Holling's “disc equation” was used:

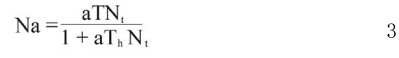

where *N_a_* is the number of prey consumed, *a* is the searching efficiency (proportion of successfully attacked prey per unit time), *T* is the total time in the patch (here, the length of the experiment was two hours or 7200 s), *N_t_* is the prey density and *T_h_* is the handling time. The parameters estimated for this equation were *a* = 0.374 ± 0.055, and *T_h_* = 182.9 ± 14.7 s (0.0253979 * 7200 s).

At the lowest prey density (1 larvae/plant), 66.6 % of *D. lineare* individuals failed to attack the prey; although, facing up to 10 larvae/plant, all earwigs ate at least 2 of them. In the type II model, as prey density increases, searching for prey becomes a less important limit on the rate of predation. Prey items are easy to locate and rate of consumption is more affected by handling time (i.e., the time it takes a predator to subdue, consume, and digest its prey). As searching becomes less important and handling becomes more important, the rate of consumption shows a decelerating rate of increase. Eventually, search is not limiting at all and the rate of consumption levels off at an upper limit determined by handling time alone (Table 1). Earwig satiation (the estimated asymptotic maximum in the model) occurred at 39.37 larvae. The observed maximum number of larvae consumed by a single female during the 2 h experiment was 42 individuals.

Many arthropod predators exhibit a type II functional response as described by Holling's disk equation; this type of response is characterized by a predation rate that is limited only by handling time. The search efficiency estimate (*a* = 0.374) obtained in this study was well within the range of others obtained for predators of Noctuidae (Lepidoptera) pest. For example, Parajulee et al. (2006) obtained 0.489 for the big-eyed bug (*Geocoris punctipes*) and 0.220 for the green lacewing (*Chrysopa oculata*) against bollworm, *Helicoverpa zea*, eggs, and Mohaghegh et al. (2001) obtained 0.067 for the pentatomid *Podisus maculiventris* preying on fourth-instar larvae of the beet armyworm, *Spodoptera exigua.* Similarly, Morales et al. (2001) reported a searching efficiency of 0.79 for the egg parasitoid *Telenomus remus* against *S. frugiperda*. Search efficiency decreases with increasing density because the predator spends more time searching for prey at lower densities (Hassel et al. 2000). Saini et al. (1997) registered a similar decrease in search efficiency when evaluating the functional response of *Podisus conexivus* attacking *Anticarsia gemmatalis* larvae.

**Table 1.**
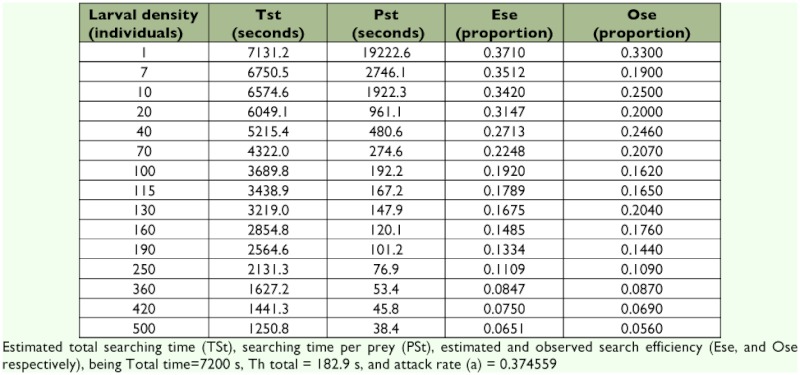
Average searching times and searching efficiencies of *Doru lineare* females

Functional response studies in small laboratory arenas have been criticized (O'Neil 1989; Wiedenmann and O'Neil 1991) because factors such as large searching areas, host plants, and weather under field conditions may influence the effectiveness of predators. Generally, the density levels used in laboratory studies are substantially higher that those occurring in the field. However, in this study, densities were realistic, taking into consideration the biology of both the predator and the pest. *S. frugiperda* eggs are deposited in layers and covered with scales from the female's body (Beserra et al. 2002). Each egg cluster has an average of 109 ± 98.6 eggs (Murua and Virla 2004), the eclosion rate is over 95 %, and the larvae remain aggregated on the host plant during the first hours after emergence. According to the findings, a single *D. lineare* female may be able to consume almost half the offspring of a single egg cluster.

An estimation of the potential impact of *D. lineare* predation on the population of *S. frugiperda* larvae may be generated by combining the results of experiments described here with estimates of earwigs and pest larvae densities *in situ*. Although the dynamics of generalist predators are not tightly coupled to those of any one of their prey, such predators can have dramatic effects on prey populations (Murdoch et al. 2002). Clearly, predation by *D. lineare* on *S. frugiperda* larvae may significantly influence survival to the larval stage in this pest. However, in functional response studies, field data are an essential complement for the laboratory results because in natural conditions other variables can interfere in predator behavior. The performance of this earwig as a potential biocontrol agent can only be appreciated when considering all relevant aspects of its biology, including development and reproduction. The present study has improved understanding of the role of *D. lineare* and its potential value in maize agroecosystems.
